# Comparative Cross-Sectional Assessment of Temporomandibular Dysfunction and Masticatory System Changes in Patients with Rheumatoid Arthritis and Temporomandibular Joint Osteoarthritis

**DOI:** 10.3390/jcm15155873

**Published:** 2026-07-27

**Authors:** Anna Wydra-Karbarz, Zbigniew Guzera, Bogdan Batko, Mateusz Moskal, Katarzyna Błochowiak

**Affiliations:** 1 Dental Office Relax Dental Spa, 26-600 Radom, Poland; anna86wydra@gmail.com; 2 Świętokrzyskie Rheumatology Center, Health Care Center, 26-200 Końskie, Poland; zetguzera@poczta.fm; 3 Department of Rheumatology and Immunology, Dietl Specialist Hospital, 31-121 Krakow, Poland; bpbatko@gmail.com (B.B.); mateusz.moskal@hotmail.com (M.M.); 4 Department of Rheumatology and Immunology, Faculty of Medicine and Health Sciences, Kraków Andrzej Frycz Modrzewski University, 30-705 Kraków, Poland; 5 Department of Oral Surgery, Poznan University of Medical Sciences, 60-812 Poznan, Poland

**Keywords:** temporomandibular joint, temporomandibular disorders, rheumatoid arthritis, osteoarthritis, masticatory system, myofascial pain, orofacial pain

## Abstract

**Background/Objectives**: Although the etiology and pathomechanism of temporomandibular joint osteoarthritis (TMJ OA) and rheumatoid arthritis (RA) are different, they can both lead to serious structural and functional changes in the masticatory system. This study aimed to assess and compare the prevalence and characteristics of functional changes in the masticatory system in RA and OA TMJ patients. **Materials and Methods**: This cross-sectional study included 40 RA patients meeting the 2010 ACR/EULAR criteria and 40 TMJ OA patients. Research diagnostic criteria for TMD (RDC/TMD) were used to assess functional changes. **Results**: Orofacial pain was a predominant TMD reported in 80% of RA patients and in 67.5% of OA TMJ patients. There was no difference in the orofacial pain intensity and degree of somatization between RA and OA TMJ patients (*p* = 0.114). Orofacial pain duration was statistically longer in OA TMJ patients than in RA patients (*p* = 0.003). The ranges of mandibular movement were limited but without any statistically significant differences between the groups compared for maximum active opening (*p* = 0.349) and maximum passive opening (*p* = 0.461). Patients with RA presented with more muscles affected with pain. The most painful muscles were the medial pterygoid muscles and the anterior belly of the digastric muscle for the right side (*p* = 0.006) and for the left side (*p* = 0.004), and the tendon of the temporal muscle (*p* = 0.011) and (*p* = 0.004). The pain intensity of these muscles was statistically higher in RA patients in comparison to OA TMJ patients. Predominant TMJ sounds were slight crepitations in both RA and OA TMJ patients, mainly during laterotrusion in OA (*p* = 0.000). **Conclusions**: Laterotrusion was the key movement differentiating TMD manifestations between the two groups. Early functional diagnosis of the temporomandibular joint and an interdisciplinary therapeutic approach, combining treatment of the underlying disease with therapy of the masticatory system disorders, are crucial to limit the progression of changes and improve masticatory function.

## 1. Introduction

The temporomandibular joint (TMJ) and the muscles of the masticatory system form a complex biomechanical unit responsible for the precise control of mastication, speech, and swallowing. Any disturbance in the TMJ and masticatory system resulting from mechanical overload or inflammatory processes can lead to significant functional and structural alterations. They can manifest as a heterogeneous group of functional limitations, symptoms and clinical features such as orofacial pain, headache, tenderness of the masticatory muscles, asymmetry in jaw movement, joint sounds, reduced mouth opening, masticatory difficulties, and altered occlusion [1,2]. According to international consensus, temporomandibular disorders (TMD) should be assessed by the Diagnostic Criteria for Temporomandibular Disorders (DC/TMD), which has substituted the former Research Diagnostic Criteria for Temporomandibular Disorders (RDC/TMD). They are based on a questionnaire and clinical examination. Research questionnaires must be translated into national languages and culturally adapted. The RDC/TMD questionnaire was intended to be merely the first step in developing a comprehensive examination and diagnostic method for TMD, suitable for use in both research and clinical practice. The RDC/TMD exhibited sufficient reliability for clinical use for the most common TMD but was incomplete in terms of prediction of disease course. A major advantage of the DC/TMD questionnaire in comparison to the former RDC/TMD protocol was the introduction and widespread adoption of radiological imaging in the diagnosis of TMD. Moreover, for the DC/TMD protocol, acceptable sensitivity and specificity levels were higher, and for a definitive diagnosis were considered as sensitivity ≥ 70% and specificity ≥ 95%. Both temporomandibular joint osteoarthritis (OA) and rheumatoid arthritis (RA) affect TMJ function. Although they use similar diagnostic tools, they may show slight differences in the diagnosis of orofacial pain, myofacial pain, arthralgia, and disk displacement with reduction and accompanying depression [3,4,5,6]. Temporomandibular joint osteoarthritis (OA) and rheumatoid arthritis (RA) are the most common disorders affecting TMJ function. Although they differ substantially in their pathomechanisms, they can produce prolonged and progressive destruction of TMJ components and surrounding structures [1,2,7,8]. Both diseases are characterized by a slow, chronic and progressive course with subsequent processes of enrolment of TMJ components and repair. Furthermore, their common starting point is the joint cartilage, whose morphological changes gradually lead to the involvement of other joint structures and affect extra-articular areas [7,8]. The inflammatory process can predispose to degenerative changes, as presented in OA, and degenerative changes may occur earlier in patients with RA or may be more severe. Osteoarthritis in the TMJ may also be a part of this generalized condition. Therefore, symptoms of both diseases can overlap and may manifest in a similar way [9,10].

Osteoarthritis (OA) is a non-inflammatory process limited to an involved joint. It represents the cumulative effect of biological and mechanical factors that, by acting on the joint, destabilize the tightly interconnected processes of cartilage degradation and regeneration as well as the remodeling of the subchondral bone. The data related to global osteoarthritis prevalence vary depending on OA definition, used methodology, and countries. It is estimated that approximately 6–7% to even 15% of the world’s population suffers from osteoarthritis, with the majority of women [10,11]. Its prevalence increases with age. It may manifest as symptomatic or asymptomatic disease detected only on radiological images. In TMJ osteoarthritis, clinical evidence of the disease is found in 8–16% of the population [10]. In the initial stage of osteoarthritis, the degenerative changes in articular cartilage are triggered by a reduced number of proteoglycans and increased water content. Ultimately, the degenerative process involves all joint components. The etiology of TMJ OA is complex, including alterations in TMJ structure, chronic overload and tension of the masticatory muscles, weakening of the periarticular muscles, postural defects, bruxism, parafunctions, malocclusion, and missing teeth. Moreover, TMJ OA differs from osteoarthritis affecting other joints. In addition to mechanical loading, pro-inflammatory cytokines and the degradation of articular cartilage play a significant role, bringing this condition even closer to RA. Pro-inflammatory cytokines, including IL-1β, TNF-α, and IL-12, present at increased levels in synovial fluid in TMJ OA patients, are associated with cartilage degradation and suppression of the synthesis of cartilage matrix through activation of matrix metalloproteinases (MMPs) and bone resorption by activation of osteoclasts by RANKL expression. Another pathological mechanism observed in TMJ OA is the activation of Notch signaling, leading to changed angiogenesis of condylar cartilage and disk [12,13].

Osteoarthritis is primarily characterized by joint pain that intensifies during loading, often leading to a marked limitation of joint mobility. As the disease progresses, joint sounds during movement, tenderness on palpation, and joint effusion may develop [9,14,15]. Prolonged compensation for joint dysfunction results in overloading the masticatory muscles. Owing to the close mechanical and neurophysiological interdependence with the temporomandibular joint, the muscles of the masticatory system undergo secondary adaptive, overload-related, or degenerative changes leading to alterations in both function and morphology of muscular structures [16].

Rheumatoid arthritis (RA) is a chronic autoimmune disease, being one of the most common connective tissue disorders. Its etiology is multifactorial, encompassing genetic predispositions, environmental triggers, and immune system dysregulation. It results from hyperplasia of the synovial lining and pannus formation, leading to progressive degradation of the cartilage matrix and subchondral bone [17,18]. Chronic inflammation of synovial joints causes destruction of joint structures and secondary changes in soft tissues. Rheumatoid arthritis affects up to 2% of the global population, with the majority of women. It begins with pain, morning stiffness, and swelling of symmetric involved joints, particularly in the hands and feet. With time, the inflammatory process can spread and affect other joints. Rheumatoid arthritis is a progressive disease, ultimately resulting in gradual joint destruction, severe disability, and multisystem involvement. Pathogenesis centers on an intra-articular inflammatory process [17,19].

The masticatory system undergoes significant alterations during the course of RA, both secondary to joint damage and as a consequence of systemic inflammation. It is estimated that over 50% of RA patients exhibit clinical manifestations of temporomandibular joint (TMJ) involvement. Assessment of TMJ involvement severity in RA can vary widely, depending on the sample group size and methodology applied. TMJ involvement in RA is often underestimated and rarely considered in clinical practice, as the TMJ, unlike other synovial joints, seldom presents with classical signs of inflammation. More commonly, patients report subjective symptoms that do not always correlate with morphological findings or imaging results [19]. Patients with RA frequently exhibit increased muscle tone, interpreted as a compensatory mechanism in response to restricted TMJ mobility and altered occlusion. Chronic joint pain and protective responses to dysfunction contribute to the development of myogeloses, myofascial trigger points, and localized increase in muscle fiber stiffness, exacerbating functional impairment and increasing the risk of secondary bruxism. Conversely, some patients demonstrate reduced muscle strength due to chronic inflammation, trophic disturbances, or secondary sarcopenia associated with inactive disease or long-term glucocorticoid therapy. Impaired coordination of muscle contractions may manifest as asymmetric mandibular movements, reduced masticatory efficiency, and muscle fatigue [20].

Both RA and TMJ OA show complex and long-term effects on the whole masticatory system. Although their pathomechanism vary, they can trigger similar joint instability and compensatory mechanisms. Although the etiology and pathomechanism of both diseases are well understood, in our opinion, there is a lack of data comparing temporomandibular joint involvement and temporomandibular dysfunction in these two conditions. It seems justified to investigate whether RA and OA are associated with the occurrence of specific functional disorders. From a clinical point of view and treatment efficacy, it is fundamental to determine the main factor determining the severity of the disease, the limitation or elimination of which is of major therapeutic importance. It seems justified to indicate the most important features characterizing TMJ and muscle involvement in RA and TMJ OA, which formulate their clinical profiles. Identification of symptoms and clinical features specific to both RA and TMJ OA may contribute to formulating more personalized therapies and preventive strategies to minimize the risk factors and structural consequences of these inflammatory conditions. The aim of this study was to present an assessment and comparison of functional changes in patients with RA and TMJ OA.

## 2. Materials and Methods

The study included 40 patients diagnosed with rheumatoid arthritis (RA)and 40 patients diagnosed with temporomandibular joint osteoarthritis (TMJ OA). All RA patients met the 2010 American College of Rheumatology/European League Against Rheumatism (ACR/EULAR) diagnostic criteria for RA [21]. All TMJ OA fulfill the diagnostic criteria for TMJ OA based on the Research Diagnostic Criteria for Temporomandibular Disorders (RDC/TMD) protocol in the Polish version [3,4,22,23]. At the time the study was designed and conducted, there was no Polish translation of the currently recommended version of the DC/TMD questionnaire. The adults who signed written informed consent and who were able to understand and complete the questionnaire and clinical assessment were enrolled in the study. RA patients were recruited from the Świętokrzyskie Rheumatology Center at the St. Luke’s Specialist Hospital in Końskie, Poland, and from the Department of Rheumatology and Immunology at the Dietl Specialist Hospital in Kraków, Poland. Patients with TMJ OA were recruited from the Dental Office Relax Dental Spa in Radom, Poland. The study was conducted between January 2023 and June 2024.

Exclusion criteria included congenital craniofacial or temporomandibular joint anomalies, previous craniofacial trauma, prior orthognathic surgery, history of TMJ disorders, and prior intra-articular TMJ injections, pregnancy and lactation. Patients wearing removable dental prostheses were also excluded.

Functional changes and disability implications in the masticatory system were assessed following the standardized I and II Axis Research Diagnostic Criteria for Temporomandibular Disorders (RDC/TMDs) in the Polish version [22,23]. A single, skilled, nonblinded practitioner evaluated current symptoms and signs of both groups through an anamnestic questionnaire and clinical examination. Demographic and clinical data were completed on the basis of patients’ medical records and patients’ history. The study was conducted according to PICO(T) framework: Population—adult patients with RA and TMJ OA, Intervention—occurrence of temporomandibular dysfunctions and changes in masticatory system assessed by the standardized I and II Axis Research Diagnostic Criteria for Temporomandibular Disorders (RDC/TMDs), Comparison—comparison of severity and intensity of temporomandibular dysfunction and masticatory system changes between RA and TMJ OA groups, Outcome—assessment of main temporomandibular dysfunction and changes in masticatory system such as maximum active opening, orofacial pain, TMJ sounds, jaw limitations, TMJ tenderness, masticatory muscles tenderness.

Sample size calculation was based on previous studies and was performed in our previous study [24]. Based on previous studies reporting 70% TMD prevalence in RA patients, we calculated that 38 participants per group would provide 80% of statistical power to detect a significant difference with α = 0.05, assuming 10% TMD prevalence in controls. Additionally, for the results in which statistically significant differences were obtained, a power analysis of the applied tests was conducted. The power of the test ranged from 0.70 to 1.

The protocol of this study was approved by the Institutional Review Board at the Poznan University of Medical Sciences (number 672/22 from 8 September 2022). Informed consent was obtained from all subjects before any study procedure was carried out. This study was performed in accordance with the ethical standards laid down in the appropriate version of the World Medical Association Declaration of Helsinki.

The calculations were carried out with Microsoft Excel 2016 and STATISTICA software (v.13 TIBCO, Palo Alto, CA, USA). Patients’ demographic data were analyzed using descriptive statistics. The Shapiro–Wilk test was used to assess the normal distribution of the variables. To examine the differences between the two groups and to verify if a normal distribution and equal variances were present, the *t*-test was used for unpaired samples; when non-equal variances were present, the Welch test was used. If normal distribution was not present, the Mann–Whitney U test was used. For qualitative variables, numbers (*n*) and proportions (%) were calculated and collected in cross tables. Categorical variables were presented in contingency tables and were analyzed using the Chi^2^ Pearson test, Fisher–Freeman–Halton test, and Fisher exact test. For normally distributed data, results were presented as mean ± standard deviation (SD), and non-normally distributed data were expressed as median (interquartile range, IQR). Bonferroni’s correction was used for multiple comparisons of TMJ pain and muscle pain between RA patients and OA TMJ and TMJ noises between RA patients and OA TMJ patients. Moreover, for some clinically relevant results, Cohen’s coefficient, Pearson’s coefficient and odds ratios with 95% confidence intervals have been calculated and presented. Differences were considered statistically significant at *p* < 0.05.

## 3. Results

### 3.1. Clinical, Demographic and Laboratory Characteristics of RA and OA TMJ Groups

Comprehensive demographic and clinical characteristics of the study participants are presented in Table 1. Patients with TMJ OA were statistically older than patients with RA (*p* = 0.016). Moreover, female gender was a predisposing factor for RA (*p* = 0.039).

### 3.2. Comparison of TMJ Involvement in RA and TMJ OA Groups

There were no statistically significant differences in TMJ involvement and TMJ complaints between TMJ OA and RA. Orofacial pain was most frequently limited to the TMJ area in both OA and RA groups. In both these groups, orofacial pain was recurrent and bilateral. It was reported by 82.5% of RA patients compared to 67.5% in the TMJ OA group. Symptoms related to the TMJ were observed in 75% of patients with RA. Palpation of the temporomandibular joint elicited pain in both the RA and TMJ OA groups and there was no difference in pain intensity between TMJ OA patients and RA patients. Mandibular movements provoked both TMJ pain and muscular pain. However, palpation triggered mainly TMJ pain in both RA and TMJ OA groups. Laterotrusion induced pain more frequently in both RA and TMJ OA groups than jaw opening and closing, and protrusion. Thorough statistical analysis with Bonferroni’s correction did not allow for the indication of differences in TMJ pain and muscle pain (*p* = 0.053), and in TMJ pain and no pain (*p* = 0.053) on the left side during right laterotrusion. The same analysis showed a statistically significant difference between muscle pain and no pain on the left side (*p* = 0.047) and between TMJ pain and muscle pain (*p* = 0.007) during left laterotrusion. Detailed data regarding temporomandibular joint and muscle pain are presented in Table 2.

There were no statistically significant differences in ranges of mandibular movement between OA TMJ and RA patients. Patients with OA TMJ presented correction of opening movement more frequently than RA patients (*p* = 0.041). Detailed data related to mandible kinematics are presented in Table 3.

### 3.3. Assessment of Myofascial Pain and Muscle Involvement in RA and TMJ OA Patients

Among patients with RA, muscle pain on palpation was reported by 42.5%, compared to 35% in the TMJ OA group. For all muscles palpated independently on both sides of the body, reported pain was significantly greater in the RA group than in the OA TMJ group. However, there was no statistically significant difference in myofascial pain between the RA and OA TMJ groups. The most painful muscles in both groups were the medial pterygoid muscles, the anterior belly of the digastric muscle, and the temporal tendon. Moreover, in the RA group, pain in these muscles was statistically greater than in TMJ OA patients. RA patients experienced pain in more muscles than TMJ OA group patients. Myofascial pain without limitation of mouth opening occurred in 35% of OA TMJ patients and in 42.5% of RA patients. In the OA TMJ group, 5% of the patients reported myofascial pain with limited mouth opening, as shown in Supplementary Table S1.

### 3.4. Self-Assessment of Degree of Somatization and Overall Health in RA and TMJ OA Patients

Self-assessment of general health was statistically worse in RA patients in comparison to the TMJ OA group (*p* = 0.030). Pain in the oral and facial areas was reported by the vast majority of participants. Orofacial pain was observed in both groups with similar frequency and similar intensity. It was mainly recurrent and of similar degree of somatization. Its impact on everyday activities was similar in RA patients and TMJ OA patients. It could be classified as moderate with a low degree of handicap. On the other hand, patients with TMJ OA usually experienced orofacial pain longer than RA patients did. More than half of the patients in both study groups reported migraine-like pain as well as pain located in the temporomandibular joint. The analysis of the degree of somatization when depressive symptoms and non-specific physical symptoms were present, both pain-related and pain-independent, showed similarity in both groups. In the study group of RA patients and TMJ OA, the most significant factors accompanying depression included: reduced energy level or psychomotor slowing, sleep disturbances, including difficulty falling asleep and early waking up, restless sleep, feeling of excessive exertion, and loss of interest in sexual activity. Detailed information on facial pain and somatization levels is presented in Table 4.

### 3.5. Assessment of TMD and Jaw Function Limitations in RA and OA Groups

**Figure 1:**
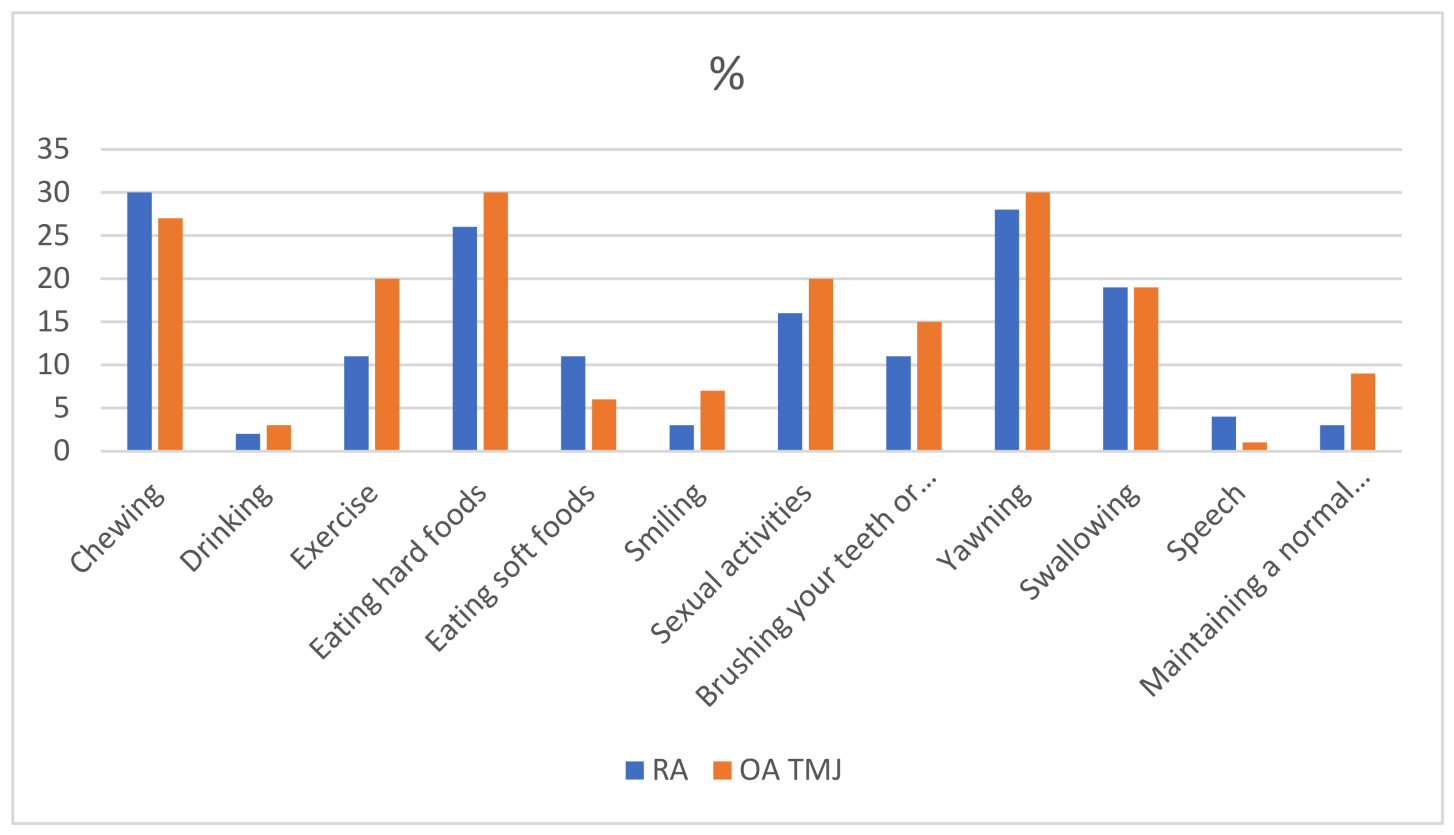
Distribution of jaw function limitations in the RA and OA TMJ group.

In the studied groups of patients with RA and TMJ OA, significant limitations in jaw function were observed. Patients most frequently reported chewing difficulties (RA—75%, OA TMJ—68%), yawning (RA—70%, OA TMJ—75%), eating hard foods difficulties (RA—65%, OA TMJ—75%), and swallowing difficulties (RA and OA TMJ—45% each). There was a statistically significant difference in exercise difficulties between RA and TMJ OA. Detailed distribution of jaw functional limitations in the group of patients with RA and OA TMJ is presented in Figure 1.

**Figure 2:**
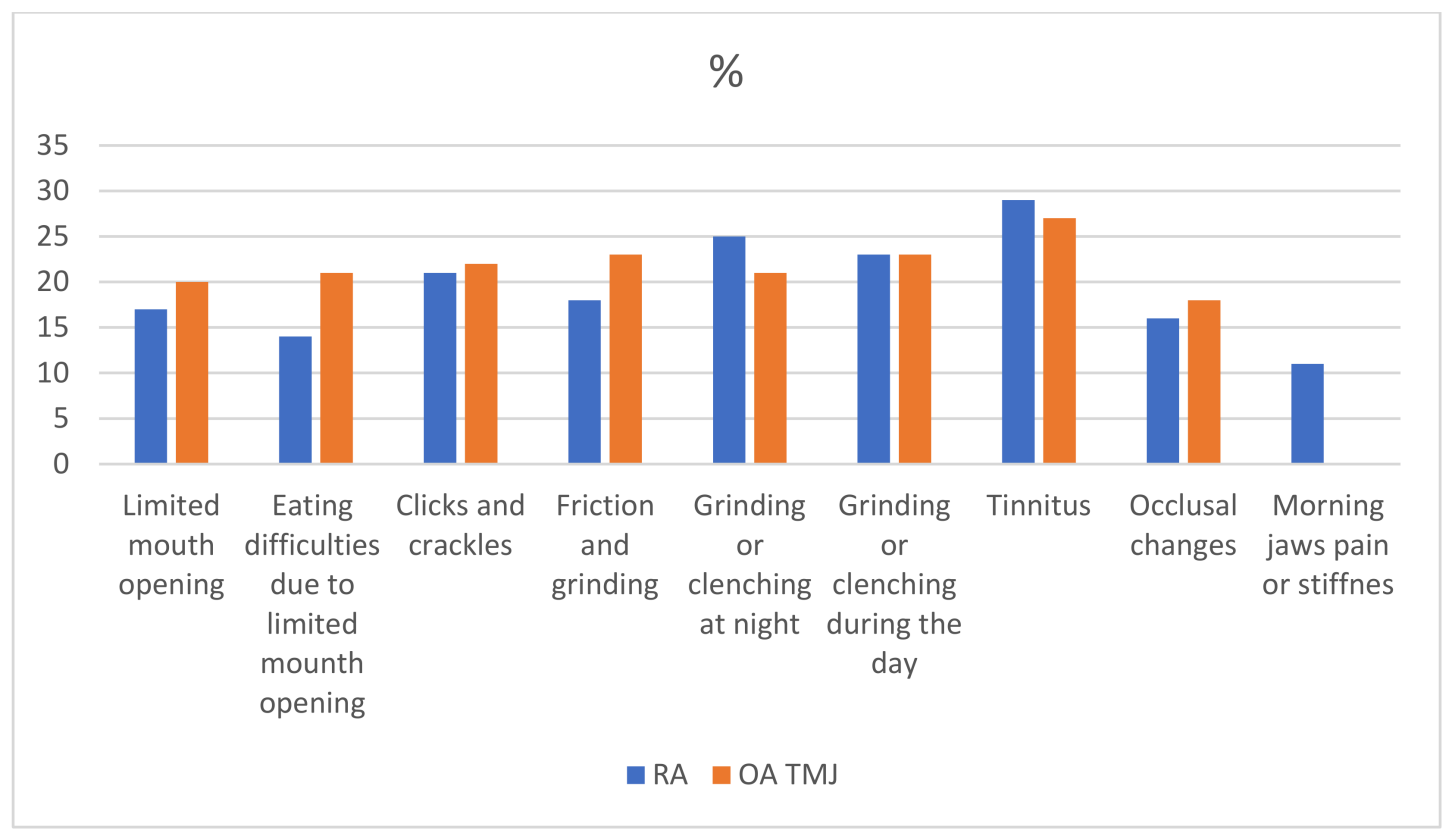
Distribution of TMD in RA and OA TMJ patients.

Among patients with RA and OA TMJ, tinnitus, teeth grinding, and clenching were the most common complaints, both during the day and at night. Temporomandibular joint (TMJ) clicks and popping sounds were also common. In the RA group, a significant proportion reported limited mouth opening and joint noise sensations, including grinding and friction. Detailed discussion of TMD reported in the RA group is presented in Figure 2.

### 3.6. Assessment of TMJ Sounds in RA and TMJ OA Groups

Mandibular movements provoked acoustic phenomena in the temporomandibular joint in both RA and TMJ OA patients. More than half of the respondents in both RA and TMJ OA groups reported acoustic symptoms in the TMJ during mouth opening and closing. Acoustic symptoms, manifested as clicking and crackles in the TMJ, were reported in 52.5% of RA patients and in 55% of TMJ OA patients. In all analyzed jaw movements, crepitus was the most frequently observed acoustic symptom. However, crackling, a specific form of acoustic symptom defined in the RDC/TMD protocol, occurred primarily during mandibular opening, showing the highest frequency in this range. Thorough statistical analysis with Bonferroni’s correction showed a statistically significant differences between clear crepitation and no TMJ sounds on the left side during opening (*p* = 0.001) and between clear crepitation and slight crepitation on the left side during closing (*p* = 0.000) and on the right side between clear crepitation and no sounds (*p* = 0.008) and between crackling and no sounds (*p* = 0.036) during right laterotrusion. There were statistically significant differences between clear crepitations and no sounds (*p* = 0.000), between crackling and no sounds (*p* = 0.027) and between slight crepitations and no sounds (*p* = 0.003) on the left side during right laterotrusion. There were statistically significant differences between clear crepitations and no sounds (*p* = 0.000) and between slight crepitations and no sounds (*p* = 0.025) on the right side during left laterotrusion. There were statistically significant differences between slight crepitations and no sounds (*p* = 0.007) and between clear crepitations and no sounds (*p* = 0.000) on the left side during left laterotrusion. The exact characteristics and frequency of sounds in the temporomandibular joint are presented in Table 5.

## 4. Discussion

The presented study found that RA and TMJ OA are characterized by a very high prevalence of various but similar functional changes and disability implications in the masticatory system according to the RDC/TMD protocol. Although both entities are caused by different factors, they can manifest in a similar way. Moreover, it seems that RA may predispose to faster development of osteoarthritis in the TMJ. Our previous study proved that RA has not induced any specific pattern of temporomandibular disorders, and the crucial role in preventing them is combating the acute stage of inflammation and reducing parameters of the inflammatory process [24]. This study indicated that patients with OA TMJ need a longer time to develop the same and similarly intense symptoms as RA patients. Patients with OA TMJ reported a statistically significantly longer history of orofacial pain than RA patients. Although our previous study did not confirm a direct relationship between RA duration and severity of TMD, it seems that OA TMJ worsens with time and may reach a similar intensity as in the course of RA, if the disease lasts long enough [24].

Orofacial pain was the predominant type of TMD in both studied groups. Its nature and features were similar. In both groups, orofacial pain was recurrent, bilateral and mainly provoked by palpation or jaw movement. It involved TMJ structures and, to a lesser extent, adjacent muscular and facial areas. It might be treated as a pain sensation of the masticatory system with intensity ranging from mild to moderate. Our findings are consistent with the previous studies that indicated the provoked and quite diffuse nature of the orofacial pain in RA and OA TMJ [19,25,26,27]. Orofacial pain is not correlated with any daily activities in both RA and OA TMJ groups and is not related to depression or any other non-physical symptom. It is hard to indicate the cause of orofacial pain in both groups. Furthermore, there were no statistically significant differences in the prevalence of migraine and myofascial pain between RA and OA TMJ. Essential likely factors contributing to pain in both groups are cervical spine and shoulder involvement. Generalized spread of arthritis in RA and quite frequent posture defects and overload of the cervical spine observed often in OA can predispose to orofacial pain. Some researchers emphasized that experiencing pain and the severity of pain could be strongly connected with the severity and the type of pathological structures observed in radiological images. [25,26,27]. On the other hand, Wu et al. revealed no correlation between TMJ pain and muscle pain and radiological findings on CBCT scans [28]. Furthermore, there was no correlation between pain severity and the TMJ overall magnetic resonance imaging scores and metalloproteinase levels assessed in the TMJ innervated tissues [29]. Comparable results were obtained by Chantaracherd et al., who found no correlations between TMJ intra-articular status and pain severity, jaw function, and disability. They suggested that TMJ intra-articular disorders have minimal impact on patients’ reported pain, function, and disability [28]. These results confirmed that pain in TMJ OA may arise from factors beyond structural changes, including functional and psychosocial impact [30]. Moreover, the intensity of pain can depend on the OA stages. Pain occurs mainly in the initial stage of OA when synovitis predominates. More advanced stages are painless and are associated with facial skeletal remodeling. Here, TMJ OA and RA are similar because pain is mainly correlated with synovitis or active stages of both diseases. Other potential factors for orofacial pain are age, gender, and accompanying osteoarthritis in other joints. Patients with TMJ OA were statistically older than those with RA. Some researchers indicated that older age and accompanying osteoarthritis may be predisposing factors for TMJ pain [31]. Moreover, pain experienced in other joints may be correlated with TMJ osteoarthritis and orofacial pain [31]. It is worth noting that laterotrusion more often triggers muscle pain than TMJ pain in both RA and OA patients. This could be a result of primary asymmetric overload of muscles or compensatory mechanisms associated with joint instability or a protective muscle behavior. In comparison to other mandibular movements, laterotrusion is more likely to produce degenerative changes observed in RA and OA TMJ. Lateral mandibular movements require precise and complex synchronization of both interconnected TMJs. Asynchronous TMJ function, especially reduced translation of the mandibular head on the balancing side, may lead to increased intra-articular tension and limited range of lateral movement. This may explain greater pain experienced during lateral mandibular movements in RA patients as well as in OA TMJ.

Our study revealed that RA and OA imply quite severe but similar changes in jaw movement. Although both RA and OA TMJ result from different causes and are associated with distinct etiopathogenesis, this does not result in significant differences or limitations regarding mandibular movements and mandibular kinematics. It seems that disease duration can be associated with more severe limitation in mandibular movement rather than the type of disease. Mouth opening and ranges of mandibular movement are decreased to a similar extent. Our previous study found a significant impairment of mouth opening in RA patients [24]. A possible explanation of the reduced ranges of mandibular movement in RA patients is intra-articular and muscular factors, including reduced capacity for extension of the capsular ligament, formation of intra-articular fibrous adhesions, or elevator muscles shortening by muscle contracture or inflammation [24,32]. On the other hand, changes in the mandibular kinematics observed in OA TMJ are caused by pain induced by the activity of the affected joint. A previous study indicated that the kinematics of OA may vary depending on the level of pain and the degree of bone degeneration [33]. Moreover, both RA and OA TMJ are progressive diseases with alternating remission and active stages, and changes in mandibular kinematics may strongly depend on the disease stage. Lower maximum mouth opening capacity could be associated with the established stage of RA and symptomatic stage of OA TMJ. Our study revealed significant functional differences in the range of mandibular opening movements. A completely unfeasible straight path of this movement for OA patients may indicate more advanced changes in TMJ biomechanics in this group. This discrepancy may result from different pathophysiological mechanisms of RA and OA TMJ. It seems that chronic inflammation observed in RA may lead to pain and temporary TMJ function disturbances, with the adaptive capacity of the musculoskeletal system partially preserved. In OA TMJ patients, permanent structural changes, such as reconstruction of articular surfaces and limited translation of the head of the mandible, favor permanent biomechanical disorders.

The presented study revealed a statistically significant difference in pain intensity of lateral and medial pterygoid muscles, anterior and posterior bellies of digastric muscles, and tendons of temporal muscles between RA and OA TMJ patients. In general, patients with RA present a greater number of painful muscles and greater intensity of pain than OA TMJ patients. It may result from the more generalized nature of RA, predisposition to myalgias, fibrositis, and ligament involvement in RA, and inflammatory mediators affecting the muscle tissue. A relatively high prevalence of several types of muscle pain in RA was detected in previous studies and ranged from 55% to 93% [33,34]. Muscle pain in RA indicates that the inflammatory process radiates beyond the temporomandibular joint. Its presence may likely be due to a high frequency of clenching and grinding of teeth as well as to tension and chronic stress accompanying the course of the generalized disease such as RA. The main source of muscle pain in OA TMJ may be degeneration of TMJ structures leading to changes in the biomechanics of the masticatory system. It triggers subsequent compensatory mechanisms in the masticatory muscles [10,35]. This compensatory mechanism develops in response to joint instability and pain. The imbalance between the activity of agonist and antagonist muscles observed in the course of OA TMJ leads to their increased resting tone. Imaging and histological reports show that compensatory hypertrophy of selected muscles may occur in the initial stages of TMJ OA, whereas in the chronic phase, secondary muscle atrophy is observed, particularly in patients with persistent pain and limited mobility. It confirms that muscle involvement may be observed even in most initial OA TMJ stages and may be correlated with TMJ pain and orofacial pain. Muscle hyperactivity leads to an increase in intramuscular tension, impaired microcirculation, and local muscle hypoxia [24]. It causes the release of inflammatory mediators and stimulation of nociceptive receptors, leading to muscle pain. Moreover, electromyographic examination revealed other changes in muscle function in OA TMJ patients, including bilateral asymmetry and abnormal motor unit recruitment patterns. Increased resting activity and impaired contraction synchronization may be a significant factor contributing to pain and limited joint mobility [36,37,38]. High intensity of pain in the medial pterygoid muscle in OA TMJ patients may be brought about by its role in stabilizing mandibular lateral movements and adaptation to asymmetric joint loads. In turn, higher intensity of pain in the temporal muscle tendon in OA TMJ patients in comparison to RA patients may result from increased compensatory activity in response to the disturbed relationship of the articular disk to the head of the mandible. It leads to the development of trigger zones and pain in the temporal area. The persistence of pathological movement patterns, such as lateral deviations or jumping movements in OA TMJ, can be both a consequence and a factor sustaining the dysfunction. Pain appears to be a central element integrating these mechanisms. In addition to its direct effect on limiting contractile force and range of motion, it induces a protective inhibition of motor neuron activation and promotes the perpetuation of abnormal activation strategies. In summary, the available data indicate that TMJ OA should be considered a condition with a complex pathophysiology, in which structural changes, biomechanical abnormalities, and neuromuscular dysfunction reinforce one another [39,40,41,42].

Our study found a high prevalence of TMJ sounds in both RA and OA TMJ. Acoustic symptoms, especially crepitations, are typical of degenerative diseases of TMJ such as osteoarthritis. They have been introduced into diagnostic criteria of OA TMJ. Their occurrence could be correlated with structural changes in TMJ observed in RA as well as in OA. Therefore, crepitations could be treated as a reliable predictor of erosion in both RA and OA. A lack of radiological examination makes assessment of potential correlation between radiological symptoms of cartilage destruction and the presence of crepitations more difficult. Some researchers indicate that crepitations are associated with RA and OA phases and predominate in their early stages, when destructive changes are the most progressive [43,44]. The predominant acoustic symptoms are slight crepitations in both RA and OA groups. However, these are lateral mandibular movements that mainly induce crepitations, with especially high prevalence in OA TMJ patients. Seemingly, RA is associated with more symmetrical TMJ involvement of both TMJs than OA is. Moreover, our study revealed that in the OA TMJ group, TMJ sounds are more severe, with predominance of clear crepitations, compared to the RA group. In RA patients, TMJ sounds are gentle, with predominant slight crepitations. Destruction of articular cartilage and TMJ structural irregularities observed in OA cause increased friction and distinct acoustic sounds. In turn, synovitis occurring in RA does not lead to such significant TMJ structural changes triggering TMJ sounds.

Limitations of the study result mainly from high heterogeneity of the patients within both groups. Our results should be verified on larger and more homogeneous groups, or all enrolled patients should be divided into subgroups for detailed analysis of the assessed symptoms and parameters. Different duration of the disease and the occurrence of symptoms make the results interpretation difficult. Both OA and RA are progressive diseases with a long medical history and changing dynamics. Some of the presented results may depend strongly on the disease stage. Furthermore, various pharmacological therapies applied in patients with RA affect the control of the active inflammatory process. It may be responsible for the onset of orofacial pain, some TMDs, and various degrees of patients’ somatization and disability. Another potential limitation of the study is that no oral health assessment was performed. In some patients, the observed symptoms may not only be caused by an underlying disease such as RA. No imaging examination, including MRI, makes it impossible to find correlations between the TMD findings observed in our study and the real structural changes in the TMJ and adjacent areas. Defining real causes of the reported symptoms is hindered, too. Finally, there was an age and gender discrepancy between OA TMJ and RA patients. Gender and age are essential confounding factors that can modify our study results. Female gender can predispose to RA and degenerative diseases, while older age predisposes to more severe degenerative changes in TMJ. Another significant limitation of the study is a lack of examiner calibration, intra-examiner reliability, and reproducibility. It could be treated as potential observer bias. Finally, the way of patient recruitment from the hospital department and private dental clinic may potentially interfere with the number of patients complaining of any TMJ problems.

## 5. Conclusions

Our findings indicated that RA could be a significant risk factor for the development of degenerative changes in the TMJ, contributing to the progression of OA and the exacerbation of TMD symptoms. These findings should be verified by radiological examination. Early functional diagnosis of the temporomandibular joint and an interdisciplinary therapeutic approach, combining treatment of the underlying disease with therapy of the masticatory system disorders, are crucial to limit the progression of changes and improve masticatory function.

## Figures and Tables

**Table 1:** Demographic and clinical characteristics of RA and OA groups.

Parameters	Patients with OA TMJ *n* = 40	Patients with RA *n* = 40	*p* Values	Effect Size	*p* Values Adjusted to Age and Gender
Age: mean ± SD, years Median (ranges), years	58.7 ± 8.5 59 (44–73)	52.6 ± 11.5 53 (27–72)	***0.016* ^1^**	0.558 *	
Gender Female/Male, *n*	*26/14*	*34/6*	***0.039* ^2^**	0.225 **	
Disease duration, years	8 (6)	6 (12.5)	*0.555* ^3^	0.133 *	*0.121*
Disease treatment duration, years	3 (4)	5 (9)	*0.182* ^3^	0.303 ^1^	***0.026***
Adjusted OR					1.08
[−95%CI; +95%CI]					[1.01; 1.16]
Family history of rheumatic diseases, *n*	*20*	*11*	*1.000* ^2^	0.225	*0.140*
OR				2.64	
[−95%CI; +95%CI]				[1.04; 6.69]	
Walking difficulties, *n*	*29*	*23*	*0.159* ^2^	0.15 **^2^	*0.348*
Concomitant diseases, *n:*	*22*	*25*	*0.667* ^2^	0.047 **	*0.491*

Unless otherwise stated, the data are expressed as medians (IQR, interquartile range); *n*, number; RA, rheumatoid arthritis; OA, osteoarthritis; SD, standard deviation; ^1^, Welch test; ^2^, Chi^2^ Pearson test; ^3^, Mann–Whitney test; *, d Cohen; **, C Pearson; OR, odds ratio.

**Table 2:** TMJ pain and muscle pain in OA TMJ patients and RA patients.

Orofacial Pain and TMJ Involvement	OA TMJ Patients *n* = 40	RA Patients *n* = 40	*p* Value	Effect Size	*p* Values Adjusted to Age and Gender
TMJ complaints yes, *n* (%)	*26* (65)	*30* (75)	*0.415* ^1^	0.091 ^2^	*0.859*
**Orofacial pain on palpation, *n*:**			*0.466* ^2^	0.176	*0.964*
**Bilateral**	*16*	*18*			
**Unilateral**	*13*	*7*			
Right side (RS)	*7*	*10*			
Left side (LS)	*4*	*5*			
**Orofacial pain on palpation, *n*:**					
***Right TMJ:***			*0.410* ^1^	0.187	*0.440*
TMJ pain	*11*	*14*			
Muscle pain	*5*	*6*			
Both TMJ and muscle pain	*8*	*11*			
None	*16*	*9*			
***Left TMJ:***		*11*	*0.398* ^2^	0.189	*0.415*
TMJ pain	*13*	*4*			
Muscle pain	*3*	*9*			
Both TMJ and muscle pain	*4*				
None	*20*	*15*			
**TMJ pain intensity on palpation:**					
***Right TMJ:***					
Lateral part	2 (1)	3 (1)	*0.107* ^3^		
Posterior part	3 (1)	3 (1)	*0.294* ^3^		
***Left TMJ:***					
Lateral part	2 (0)	2 (1)	*0.215* ^3^		
Posterior part	2 (1)	2 (1)	*0.302* ^3^		
**Orofacial pain during jaw movement on right/left side RS/LS, *n*:**					
***during maximum active opening:***					
TMJ pain	*14/17*	*19/17*			
Muscle pain	*13/13*	*9/12*			
Both TMJ and muscle pain	*9/4*	*6/5*	*0.548* ^2^/*< 1* ^2^	0.164/0.043	*0.204/0.532*
None	*4/6*	*5/6*			
***during maximum passive opening:***					
TMJ pain	*18/13*	*19/15*			
Muscle pain	*15/17*	*11/12*			
Both TMJ and muscle pain	*3/2*	*3/0*	*0.690* ^2^/*0.272* ^2^	0.134/0.223	*0.536/0.158*
None	*4/8*	*7/13*			
**Right laterotrusion right RS/LS, *n*:**					
TMJ pain	*14/18*	*9/6*			
Muscle pain	*19/17*	*21/24*			
Both TMJ and muscle pain	*3/1*	*3/1*	*0.578* ^2^/***0.016*** **^2^**	0.156/0.320	*0.954/0.280*
None	*4/4*	*7/9*			
**Left laterotrusion left RS/LS, *n*:**					
TMJ pain	*16/16*	*8/6*			
Muscle pain	*19/12*	*23/28*			
Both TMJ and muscle pain	*2/3*	*6/3*	*0.176* ^2^/***0.002*** **^2^**	0.244/0.385	*0.058/**0.007***
None	*3/9*	*3/3*			
**Protrusion RS/LS, *n:***					
TMJ pain	*21/21*	*20/17*	*0.871* ^2^/*0.500* ^2^	*0.101/0.170*	*0.215/0.216*
Muscle pain	*9/12*	*8/11*			
Both TMJ and muscle pain	*6/3*	*9/3*			
None	*4/4*	*3/9*			

Unless otherwise stated, the data are expressed as medians (IQR interquartile range); ^1^, Chi^2^ Pearson test; ^2^, Fisher–Freeman–Halton test; ^3^, Mann–Whitney test.

**Table 3:** Characteristics of mandibular kinematics.

Parameter	OA TMJ Patients *n* = 40	RA Patients *n* = 40	*p* Value	Effect Size	*p* Values Adjusted to Age and Gender
Maximum active opening, [mm]	34 (3)	33.5 (4.5)	*0.349* ^1^	*0.208* ^1^	*0.486*
Maximum active opening without pain [mm]	32 (5)	31 (5.5)	*0.911* ^1^	*0.208* ^1^	*0.572*
Maximum passive opening [mm]	34 (3)	34 (5)	*0.461* ^2^	*0.166* ^1^	*0.393*
Incidences of limited jaw opening, *n*	*20*	*17*	*0.501* ^3^	*0.075* ^2^	*0.467*
Limited ability to eat, *n*	*21*	*14*	*0.214* ^4^	*0.229* ^2^	*0.067*
Midline deviation Right/Left, *n*	*23/17*	*18/22*	*0.262* ^3^	*0.124* ^2^	*0.155*
Incisor 11 involvement, *n* Incisor 21 involvement, *n*	*23* *17*	*24* *16*	*0.820* ^3^	*0.025* ^2^	*0.523*
Abduction, *n*:			***0.041*** **^5^**	0.325 ^2^	***0.032***
Simple	*0*	*5*			
Left corrected	*12*	*16*			
Right corrected	*20*	*10*			
Left uncorrected	*5*	*7*			
Right uncorrected	*3*	*2*			

Unless otherwise stated, the data are expressed as medians (IQR interquartile range); ^1^, Mann–Whitney’s test; ^2^, *t*-test; ^3^, Chi^2^ Pearson’s test; ^4^, Fisher’s exact test; ^5^, Fisher–Freeman–Halton’s test.

**Table 4:** Comparison of orofacial pain and degree of somatization between the RA and TMJ OA group.

Parameter	TMJ OA	RA	*p* Value	Effect Size	*p* Value Adjusted to Age and Gender
Self-assessment of general health	4 (1)	4 (2)	***0.030*** **^1^**	0.502	0.075
Self-assessment of oral health	3 (1)	3 (1)	*0.414* ^1^	0.183	*0.550*
Pain or swelling of joints excluding TMJ, *n*	*33*	*39*	*0.057* ^3^	0.243	*0.072*
**Orofacial pain**					
Chronic orofacial pain yes, *n* (%)	*27* (67.5)	*32* (80)	*0.204* ^2^	0.141	*0.597*
Facial and orofacial pain characteristics:			*<1* ^3^	0.035	*0.665*
Constant pain, *n*	*3*	*3*			
Recurrent pain, *n*	*19*	*24*			
Single incidental pain, *n*	*5*	*6*			
Chronic Orofacial Pain duration, years	3 (3)	2 (2.3)	***0.003*** **^1^**	0.810	***0.029***
Chronic Orofacial Pain intensity	70 (10)	66.7 (10)	*0.114* ^1^	0.413	*0.090*
Number of days with impaired mobility, *n:*				0.375	***0.001***
Ranges	0–9 days	3–10 days			
0–6 days	*37*	*23*			
7–14 days	*3*	*17*			
15–30 days	*0*	*0*			
≥31 days	*0*	*0*			
Pain impact on everyday life, *n:*			*0.104* ^3^	0.279	***0.029***
0	0	2			
1	0	1			
2	11	20			
3	16	12			
Chronic pain classification:			*0.582* ^3^	0.166	*0.305*
I low intensity, *n* (%)	*0* (0)	*2* (5.88)			
II high intensity, *n* (%)	*10* (37.04)	*13* (38.24)			
III moderate, *n* (%)	*17* (62.96)	*19* (55.88)			
IV severe, *n* (%)	*0*	*0*			
Handicap assessment:			*0.871* ^2^	0.071	0.525
Low, *n*	10	15			
High, *n*	17	19			
Migraine, yes, *n*	*22*	*21*	*0.822* ^2^	0.025	*0.178*
Depression	1.32 (0.9)	1.42 (0.9)	*0.106* ^1^	0.367	*0.087*
Non-specific physical symptoms (including pain-related symptoms)	2 (1.16)	2 (0.83)	*0.473* ^1^	0.161	*0.617*
Non-specific physical symptoms (without pain-related symptoms)	1.9 (1.21)	2 (0.93)	*0.881* ^1^	0.033	*0.892*

Unless otherwise stated, the data are expressed as medians (IQR interquartile range); ^1^, Mann–Whitney test; ^2^, Chi^2^ Pearson test; ^3^, Fisher–Freeman–Halton test.

**Table 5:** Comparison of TMJ noises between RA patients and TMJ OA patients.

	RA Patients *n* = 40	TMJ OA Patients *n* = 40	*p* Value	Effect Size	*p* Value Adjusted to Age and Gender
Clicking or crackles during opening/closing, *n* (%)	*18* (45)	*23* (57.5)	*0.263* ^1^	0.025	*0.830*
**TMJ sounds during:**					
**Opening for RS/LS, *n:***					
Clear crepitation	*10/7*	*9/20*	*0.780* ^2^/***0.001*** **^2^**	0.118/0.390	*0.294/**0.001***
Slight crepitation	*18/15*	*15/11*			
Crackling	*8/4*	*12/6*			
None	*4/14*	*4/3*			
**Closing for RS/LS, *n*:**					
Clear crepitation	*10/8*	*15/18*	*0.315* ^2^/***0.000*** **^2^**	0.210/0.457	*0.072/**0.001***
Slight crepitation	*16/7*	*12/14*			
Crackling	*7/3*	*10/5*			
None	*7/22*	*3/3*			
**Right laterotrusion for RS/LS, *n:***					
Clear crepitation	*6/4*	*14/14*	***0.005*** **^1^**/***0.000*** **^2^**	0.370/0.461	***0.001/0.000***
Slight crepitation	*17/16*	*15/20*			
Cracking	*3/1*	*8/4*			
None	*14/19*	*3/2*			
**Left laterotrusion for RS/LS, *n*:**					
Clear crepitation	*4/5*	*16/16*	***0.000*** **^2^**/***0.000*** **^2^**	0.423/0.443	***0.002/0.000***
Slight crepitation	*16/15*	*19/18*			
Cracking	*3/2*	*2/4*			
None	*17/18*	*3/2*			
**Protrusion for RS/LS, *n:***					
Clear crepitation	*11/7*	*10/9*	*0.607* ^2^/*0.325* ^2^	0.158/0.206	*0.165/**0.029***
Slight crepitation	*19/25*	*20/24*			
Cracking	*5/2*	*8/5*			
None	*5/6*	*2/2*			

^1^, Chi^2^ Pearson test; ^2^, Fisher–Freeman–Halton test.

## Data Availability

The original contributions presented in this study are included in the article/Supplementary Material. Further inquiries can be directed to the corresponding author.

## Supplementary Materials

The following supporting information can be downloaded at: https://www.mdpi.com/article/10.3390/jcm15155873/s1, Table S1: Myofascial pain and muscle involvement in RA and TMJ OA patients.
