# Supplementary material for: Comparative Cross-Sectional Assessment of Temporomandibular Dysfunction and Masticatory System Changes in Patients with Rheumatoid Arthritis and Temporomandibular Joint Osteoarthritis
Source: J Clin Med. 2026 Jul 27;15(15):5873. doi: 10.3390/jcm15155873 (PMC13466788; doi:10.3390/jcm15155873)
Supplement: Supplementary file 1 [file jcm-15-05873-s001.zip › jcm-4393793-supplementary.pdf]

Supplementary Materials

Table S1. Myofascial pain and muscle involvement in RA and TMJ OA patients.

	**OA TMJ patients n=40**		**RA patients n=40**		***P value***		**Effect size**		***P* values adjusted to Age and Gender**	
	Right side	Left side	Right side	Left side	Right side	Left side	Right side	Left side	Right side	Left side
**Temporal muscle**										
Anterior	3 (1)	2 (0)	3 (1)	2 (1)	*0.057*[1]	***0.038***[1]	0.435	0.476	*0.122*	*0.117*
Central	2 0.5)	2 (0)	2 (0)	2 (0)	*0.076*[1]	***0.022***[1]	0.404	0.527	*0.455*	*0.181*
Posterior	2 (0)	2(0.5)	2 (0)	2 (0)	*0.163*[1]	***0.030***[1]	0.316	0.499	*0.509*	*0.114*
**Masseter muscle:**										
Superior	2 (1)	2(0.5)	2.5 (1)	2(0.5)	*0.086*[1]	*0.377*[1]	0.391	0.198	*0.102*	*0.718*
Central	2 (1)	2 (1)	3 (1)	2 (1)	***0.031***[1]	*0.251*[1]	0.506	0.264	*0.051*	*0.203*
Inferior	2(0.5)	2 (0)	2 (0.5)	2 (0)	*0.496*[1]	***0.034***[1]	0.153	0.486	*0.464*	*0.080*
**Stylohyoid muscle and posterior belly of digastric muscle**	2 (0)	2 (0)	3 (1)	2 (1)	***0.003***[1]	***0.000***[1]	0.695	**0.882**	***0.012***	***0.001***
**Submandibular region (Medial pterygoid muscles and anterior belly of digastric muscle)**	2(1.5)	2(0.5)	3 (1)	3 (1)	***0.006***[1]	***0.004***[1]	0.643	0.665	***0.016***	***0.009***
**Intraoral muscles:**										
**Lateral pterygoid muscle area (behind the upper molars)**	2 (0)	2 (0)	2 (1)	2 (1)	***0.004***[1]	***0.002***[1]	0.676	0.708	***0.007***	***0.009***
**Tendon of temporal muscle**	3 (1)	2 (2)	3 (0)	3 (1)	***0.011***[1]	***0.004***[1]	0.586	0.668	0.061	**0.024**
**Total number of painful muscles, *n* (%):**										
Ranges	*13-19* (47.5- 100)		*14–20* (70–100)		*0.213*		0.281		*0.948*	
**Myofascial pain, *n* (%):**	*14* (35)		*17* (42.5)		*0.571*[2]					
**Myofascial pain with limited mouth opening, *n* (%):**	*2* (5)		*0* (0)		*0.571*[2]					

Unless otherwise stated, the data are expressed as medians (IQR interquartile range); [1], Mann-Whitney test; [2], Fisher-Freeman-Halton test.
